# Loretine

**Published:** 1894-04-14

**Authors:** 


### LORETINE.

Schinzinger[1] has brought this substance before the profession as an antiseptic. It is a variety of quinoline. It is used chiefly as a substitute for iodoform, which it closely resembles. It is a yellow crystalline powder, quite inodorous, and sparingly soluble in water, alcohol, ether, and oils. It may be employed in the pure state, or mixed with calcined magnesia, in which case it forms an excellent dusting powder for wounds. The sodium salt of loretine is soluble in water, giving an orange-coloured solution. In the strength of 2 to 5 per cent. it may be used instead of carbolic acid lotion for all the purposes for which the latter is employed. The compound of calcium with loretine is used in the preparation of loretine gauze, which is made by saturating ordinary gauze in a solution of sodium loretine, and then placing it in a solution of calcium chloride, when calcium loretine is precipitated in the interstices of the gauze. Loretine wool is prepared by impregnating wool with a solution of loretine in collodion. The powder, the lotion, the gauze, and the wool are used as ordinary antiseptic dressings. Under the influence of loretine, wounds heal by first intention without fever. It has absolutely no toxic action, it does not irritate the skin, and never causes erythema or eczema; indeed, it is an excellent application for eczema, and is also very useful in lupus; in the latter disease the skin is freely cauterised, and then treated with loretine collodion. It has also been used with great advantage in the treatment of boils and erysipelas.

[1] La Semaine Médicale. December, 1893.

